# Hepatocellular carcinoma cells induce γδ T cells through metabolic reprogramming into tumor-progressive subpopulation

**DOI:** 10.3389/fonc.2024.1451650

**Published:** 2024-09-06

**Authors:** Jinkun Xia, Chaoyu Wang, Biao Li

**Affiliations:** Department of Vascular and Thyroid Surgery, Guizhou Provincial People’s Hospital, Guiyang, Guizhou, China

**Keywords:** γδT cells, hepatocellular carcinoma, fatty acid, tumor immune microenvironment, metabolic reprogramming

## Abstract

Tumor immune microenvironment (TIME) is a tiny structure that contains multiple immune cell components around tumor cells, which plays an important role in tumorigenesis, and is also the potential core area of activated immunotherapy. How immune cells with tumor-killing capacity in TIME are hijacked by tumor cells during the progression of tumorigenesis and transformed into subpopulations that facilitate cancer advancement is a question that needs to be urgently addressed nowadays. γδ T cells (their T cell receptors are composed of γ and δ chains), a unique T cell subpopulation distinguished from conventional αβ T cells, are involved in a variety of immune response processes through direct tumor-killing effects and/or indirectly influencing the activity of other immune cells. However, the presence of γδ T cells in the tumor microenvironment (TME) has been reported to be associated with poor prognosis in some tumors, suggesting that certain γδ T cell subsets may also have pro-tumorigenic effects. Recent studies have revealed that metabolic pathways such as activation of glycolysis, increase of lipid metabolism, enhancement of mitochondrial biosynthesis, alterations of fatty acid metabolism reshape the local TME, and immune cells trigger metabolic adaptation through metabolic reprogramming to meet their own needs and play the role of anti-tumor or immunosuppression. Combining previous studies and our bioinformatics results, we hypothesize that γδT cells compete for resources with hepatocellular carcinoma (HCC) cells by means of fatty acid metabolic regulation in the TME, which results in the weakening or loss of their ability to recognize and kill HCC cells through genetic and epigenetic alterations, thus allowing γδT cells to be hijacked by HCC cells as a subpopulation that promotes HCC progression.

## Introduction

Hepatocellular carcinoma (HCC) often originates from viral infection or chronic inflammation due to fatty liver (1). The immune microenvironment of HCC is highly heterogeneous due to the complex regulation of multiple factors such as genetic, viral and environmental conditions (2). These differences in immune composition and functional characteristics not only determine the progression of HCC, but also influence the patient’s response to treatment (3). Immune-checkpoint blockade (ICB) is an efficient way to restore the ability of tumor-infiltrating T lymphocytes to clear malignant tumors, but it is only less than 20% effective for HCC patients (4). One important reason for this low response rate is that tumors remodel their microenvironment in ways that promote the exhaustion and inactivation of effector T cell, thereby leading to “immune escape” (5). For example, CD8+ T cells initially infiltrate tumors and specifically recognize tumor antigens in order to initiate tumor killing ability. However, tumor cells can counter this by contributing to the formation of a variety of immunosuppressive tumor microenvironments (TME). These can limit the infiltration, activation and cytotoxicity of CD8+ T cells by suppressing IFN signaling, repressing chemokine production, and increasing the expression of co-inhibitory molecules such as PD-L1 (6). Therefore, clarifying the composition of the immune microenvironment of HCC and its response network is particularly important for finding potential targets and developing novel and effective drugs for HCC.

The activation of αβT cells is traditionally characterized by recognition of peptides originating from proteins that are expressed in a cell and then presented in specific human leukocyte antigen (HLA) molecules. Since each individual has their own group of HLA molecules, the αβT cells-activated immune response cannot be easily transferred between individuals, which limit their applications into the clinical settings (7). In contrast, the non- major histocompatibility complex (MHC) -restricted feature of γδT cells determines that they can exert their function of directly recognizing and binding a wide range of antigens without antigen presentation, suggesting that these cells could also exhibit antitumor activity with low mutational burdens and decreased MHC (8). The T cell receptors (TCRs) of γδT cells is composed of two chains, γ and δ, and human γδT cells selectively express 6 functional Vγ genes, including Vγ 2、Vγ 3、Vγ 4、Vγ 5、Vγ 8、Vγ 9 and 8 types of Vδ genes, sorted into Vδ 1-8 according to the difference of δ chains (9). Several studies have confirmed the protective role of γδ T cells in transplantable or spontaneous cancer models, and their antitumor function is closely associated with their production of interferon γ (IFNγ) and tumor necrosis factor (TNF), and/or their cytotoxic potential (7). Current studies have revealed that the T cells responding to immunotherapy actually enter the tumor from the peripheral blood after immunotherapy, rather than the T cells originally present in the tumor (10), suggesting that immune cells in the TME where contact with tumor cells occurs have a distinct molecular phenotype from those in the periphery. It is still unknown whether the γδT cells acting in the tumor are circulating infiltrated cells or resident cells that expand within the transformed tissue. In addition, most γδ T cells in premalignant or nontumor colons exhibit cytotoxic markers, whereas tumor-infiltrating γδ T cells express a protumorigenic profile (11–13). Whether this phenomenon suggest that γδ T cells in the TME may undergo epigenetic regulations or gene rearrangements that allow them to generate alternative molecular phenotypes needs to be further explored.

Cancers alter pathways of nutrient metabolism to meet the bioenergetic, biosynthetic, and redox demands of malignant cells, and these reprogrammed activities are now considered hallmarks of cancer (14). Correspondingly, immune cell metabolism is an emerging area of research aimed at elucidating the impact of key metabolic pathways on immune cell development, proliferation and function (15). The immune system continuously senses and responds to external environmental stimuli, which is a process of enormous energy demand and consumption. Innate or adaptive immune cells secrete large amounts of cytokines, chemokines, and inflammatory mediators upon activation; these immune responses are dependent only on the rapid uptake and utilization of glucose, amino acids, and fatty acids by immune cells from the microenvironment. Recent studies have pointed out that a key step in the activation of extracellular signals-induced maturation of immune cells is the reorganization of their cellular metabolism (16). The reprogramming of energy metabolism has historically been considered the basis for immune cells to perform specific functions, and cellular metabolism is one of the important mechanisms regulating the innate and adaptive immune responses. However, immune cells usually lack nutritional reserves, which drives us to consider the following question: Is the tumor-killing capacity of immune cells related to their nutritional reserves? How do immune cells compete with tumor cells for nutrients in a TME where tumor cells are highly proliferative? Does the process of nutrient competition drive the immune cells’ genetic reprogramming or epigenetic regulation, and does those alterations change their anti-tumor activity?

## Materials and methods

The transcription data GSE38476 were downloaded from the Gene Expression Omnibus (GEO) database (http://www.ncbi.nlm.nih.gov/geo/). Differentially expressed genes (DEGs) among HCC-derived and paired peritumor-derived γδT cells were displayed in the form of volcano plots by the “gplot” R package from Assistant for Clinical Bioinformatics (https://www.aclbi.com/static/index.html#/) platform. |Fold Change| > 2 and adjusted p<0.05 were set as the statistical threshold value for differentially expressed genes. Gene Ontology (GO) and Kyoto Encyclopedia of Genes and Genomes (KEGG) pathway enrichment analyses were performed by the Assistant for Clinical Bioinformatics platform using ClusterProfiler package in R software. In the enrichment result, p < 0.05 is considered to be a meaningful pathway.

## Justification of the hypothesis

### Enrichment analysis of HCC infiltrating γδT cells-related differentially expressed genes

**Figure 1:**
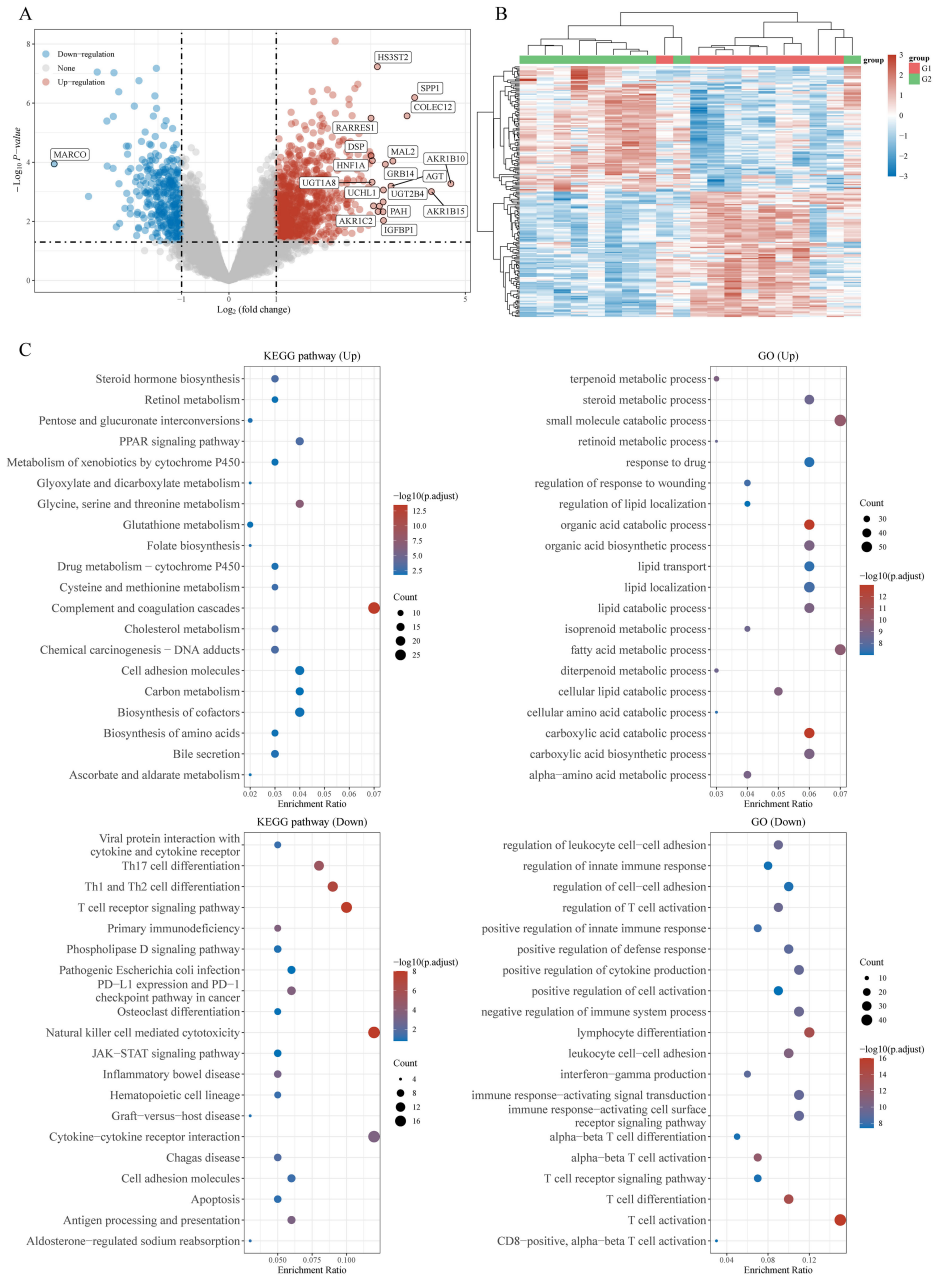
Potential biological mechanisms of HCC infiltrating γδT cells-related differentially expressed genes (DEGs). **(A)** Volcano map of DEGs, **(B)** heatmap of DEGs, and **(C)** the Gene Ontology (GO) and Kyoto Encyclopedia of Genes and Genomes (KEGG) analyses for high and low γδT cells-related DEGs based on the TCGA dataset.

Gene Expression Omnibus (GEO) is a public repository for high-throughput microarray and next-generation sequence functional genomic data sets. We downloaded and extracted the transcriptome data and corresponding clinical information of GSE38476, where γδ T cells were positively selected and the differentially expressed genes (DEGs) between HCC-derived and paired peritumor-derived γδT cells were identified, to instigate the potential regulatory mechanism of γδT cells during HCC progression, and carried out enrichment analysis as described before (17). Interestingly, we found that the upregulation of DEGs is mainly related to lipid metabolism pathways, including lipid transport, regulation of lipid localization, lipid metabolic process, fatty acid metabolic process, etc; while downregulation of DEGs is closely associated with regulation and activation of immune cells, such as regulation of T cell activation, regulation of innate immune response, positive regulation of defense response, positive regulation of cytokine production, lymphocyte differentiation, IFNγ production (the main mediator of γδT cell mediated antitumor effects), T cell receptor signaling pathway, etc (**Figure 1**). The results of enrichment analysis seem to give us some suggestion that there is a certain deviation between immune activation and lipid metabolism. Interestingly, different subgroups of γδT cells have different metabolic requirements. For example, IFN-γ+ γδ T cells were almost exclusively dependent on glycolysis, whereas IL-17+ γδ T cells strongly engaged oxidative metabolism, with increased mitochondrial mass and activity (18). This suggests that the subgroups of γδT cells in different molecular phenotypes have different metabolic programs during HCC progression. In the following sections, we will explore this phenomenon and try to offer some answers.

### Cancer cells adapt to hypoxic microenvironment through metabolic reprogramming

The tumor-killing activity of immune cells determines the anti-tumor effect of immunotherapy; hence, how to synergize with and optimize immune-checkpoint blockade (ICB) therapies have become a current research hotspot. In the tumor immune microenvironment, immune receptors, signal proteins and transcription factors will promote the activation of T cells; on the other hand, changes in metabolic pathways will affect the survival, proliferation, differentiation, function and other important biological processes of T cells. Most patients did not benefit from immunotherapy, because some immune cells were hijacked by the TME, making them lose their tumor killing ability or turn into promoters of tumor progression (19, 20). The reasons for this phenomenon is tumors utilize several immunological processes, such as targeting regulatory T-cell function or their secretory products, antigen presentation, altered production of immunosuppressive mediators, tolerance, and immune deviation allowing many factors to contribute to immunosuppressive TAM persistence despite having a functional immune system (21–23). In summary, cancer cells build an immunosuppressive microenvironment to block the attack of T cells.

Rapid proliferation and invasion of tumor cells and insufficient blood supply of local tumor tissue result in a hypoxic state of TME. To adapt to the hypoxic microenvironment and improve metabolic adaptation, some tumor cells solve the survival pressure by changing the energy mode, as shown by the increased aerobic glycolysis and impaired oxidative phosphorylation (aerobic glycolysis, Warburg effect) (24). With the gradual increase of glycolysis level, reactive oxygen species (ROS) and lactic acid accumulate in the tissues, and ROS stabilize HIF-1α (a transcription factor with a pivotal role in physiological and pathological responses to hypoxia) expression and promote the continuous occurrence of glycolytic reprogramming (25). Meanwhile, cancer cells recruit, regulate and hijack adjacent non-malignant cells, including fibroblasts, immune cells and non-cellular components, by secreting cytokines, metabolites and other biochemical molecules, to provide energy for their continuous and uncontrolled growth, invasion and metastasis (26). This implies that T cells are in nutritional competition with tumor cells in a hypoxic TME.

### Differentiation of immune cells require dynamic reprogramming of cellular metabolism

Activation of immune cell requires large amounts of energy and metabolic intermediates to meet the biosynthetic requirements for proliferation, differentiation, and execution of effector functions. The metabolic pattern of activated immune cells is very different from that of non-activated immune cells, which is very similar to the growth of tumor cells, i.e., the phenomenon of “metabolic reprogramming” (27–29). Meanwhile, the phenotype and function of immune cells are regulated by metabolic process. However, the understanding of the metabolic mechanisms of immune cells and their function is not yet well understood. Currently, the recognized metabolic patterns of immune cells can be simply divided into three categories: (1) Activated effector T cells and effector B cells use glycolysis to produce energy; (2) M1 macrophages, activated neutrophils, and dendritic cells (DCs) mainly use glycolysis to produce ATP to maintain cellular functions and this process without oxidative phosphorylation (OXPHOS) (30); (3) Quiescent immune cells, such as regulatory T cells (Treg) and M2 macrophages, generally use the tricarboxylic acid cycle to generate ATP coupled with oxidative phosphorylation to maintain cellular function, and fatty acid oxidation is also highly active in these cells (31). Different metabolic patterns also affect the differentiation of different T cell subsets. For example, Treg cells primarily utilize oxidative phosphorylation and mitochondrial fatty acid oxidation, whereas Th17 cells require glycolysis to growth and survival (32). In addition, acetyl-CoA carboxylase 1 (ACC1) mediated *de novo* fatty acid synthesis promotes Th17 cell differentiation while inhibiting Treg cells formation (33).

In summary, immune cells in different activation states or differentiation stages exhibit different metabolic patterns, and this active selection of metabolic pathways allows immune cells to adapt to their functional requirements; on the other hand, the metabolic state of the organism affect the phenotype and function of immune cells. How metabolic adaptation determine functional specialization of immune cells is fundamental to our understanding and therapeutic modulation of the immune system.

### Cancer cells change T cell activity and phenotype by modulating metabolic pathways

Cellular metabolism has emerged as a crucial determinant of viability and functionality of both cancer cells and immune cells in TME. Tumor cells reprogram their metabolism to produce specialized metabolites that provide fuel for their own growth and allow tumor immune evasion (35). For example, excessive fumarate, ammonia, linoleic acid, and cholesterol biosynthesis intermediate lanosterol generated by tumor cells can accumulate in the TME, suppress the infiltration and activation of CD8+ T cells and thus minimize their antitumor effects (36–38). Metabolic conditions in the TME are influenced by various factors, including gradients of nutrients and metabolic interactions between cancer cells and stromal cells. For instance, the anabolic metabolism of immune cells in TME also relies on glucose except for the aerobic glycolysis process observed in cancer cells (34). At present, the hotspots of metabolic reprogramming of immune cells in cancer progression mainly focuses on glucose metabolism, lipid metabolism, tricarboxylic acid cycle and amino acid metabolism, which affect the function of many immune cells by a series of key metabolic signaling pathways, such as PI3K/AKT, mTOR, AMPK, HIF-1α, c-Myc and p53 (39). This convergence of metabolic adaptations leads to a fundamental competition for nutrients between cancer cells and immune cells within the TME. The nutrient competition between tumor cells and immune cells weaken the anti-tumor function of immune cell, and even force a shift in their phenotype. For example, when myeloid cells infiltrated into the HCC microenvironment are defeated by cancer cells in competition for glutamine, their own endoplasmic reticulum homeostasis is disrupted, which leads to up-regulation of IRE1α/XBP1 signaling-induced expression of the G-protein-coupled receptor GPR109A, and myeloid cells thus turn into cancer accomplices with highly immune-suppressing properties (40). The liver plays a key role in maintaining overall metabolic homoeostasis by regulating the uptake of glucose, lipid and amino acid, and the reprogramming of metabolic processes serves as the driving force behind the initiation and advancement of hepatic malignancies. However, the underlying mechanisms of how these metabolic requirements affect immunosurveillance in TME, and consequently hinder or promote the development of HCC remains largely unknown. γδ T cells influence the recruitment of neutrophils and macrophages, and regulate many immune responses through the production of proinflammatory cytokines in cancer development. γδT1 cells, which produce IFN-γ, mainly play an anticancer function, whereas Il-17-secreting γδ T cells are considered mainly protumor effector due to the induction of angiogenesis and cancer cell proliferation (41). Although there is no direct evidence revealing the existence of nutrient competition between HCC cells and γδ T cells in the TME and their involvement in the process of inducing pro-cancer molecular phenotypes in γδ T cells and constructing an immunosuppressive microenvironment, in conjunction with the above studies, we hypothesize that the energetic demands of rapid HCC progression need to be met by HCC cells metabolic reprogramming. Elucidating the mechanisms by which HCC and their various products can suppress immune cell infiltration and activation, and the alterations in γδ T cells activity, infiltration levels, and molecular phenotypes in TME, are essential for improving therapy-directed immune responses.

### Metabolic reprogramming of T cells affects their molecular phenotype

**Figure 2:**
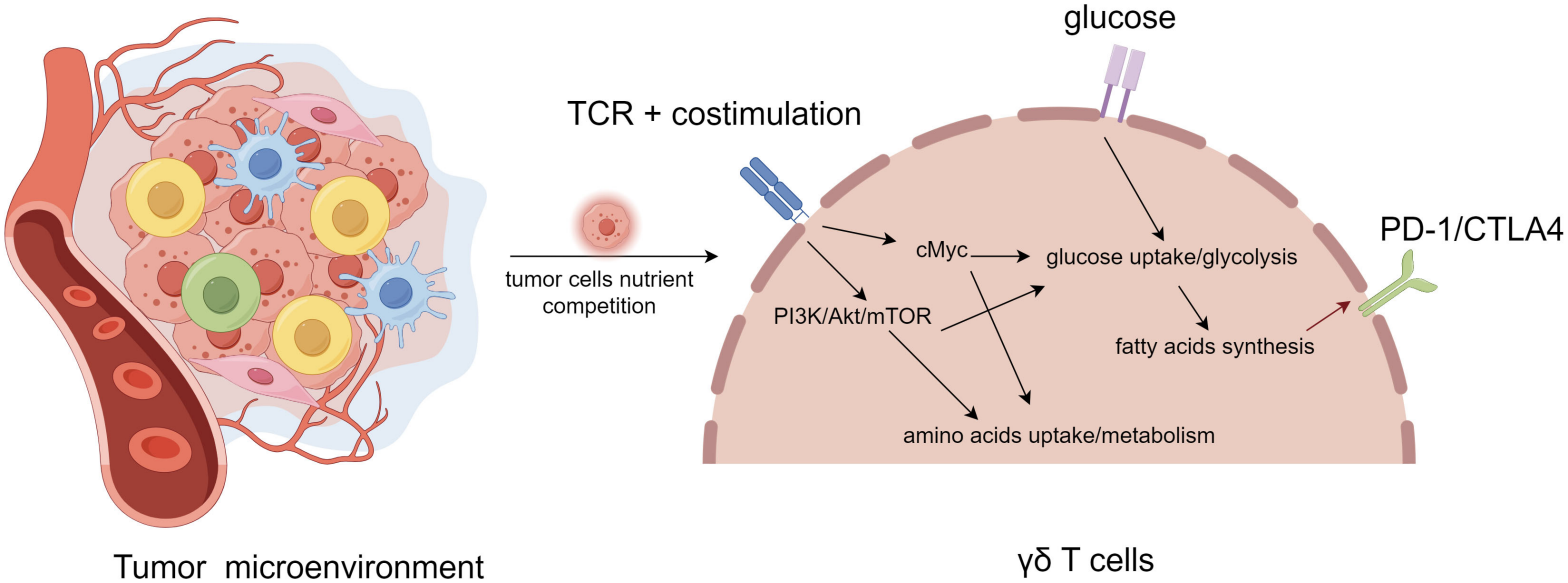
Metabolic regulation and feedback of γδT cell in tumor microenvironment. γδT cell activation and signaling through T cell receptor (TCR) activates PI3K/Akt/mTOR and cMyc pathways, leading to increased glycolysis and metabolism, decreased fatty acid oxidation, without obvious changes in oxidative phosphorylation levels. With the extremely rapid expansion of cancer cells, T cells undergo metabolic reprogramming in order to survive by means of fatty acid metabolism, resulting in altered molecular phenotypes (e.g., PD1, CTLA4), thus becoming a subpopulation of tumor-promoting progression T cells.

Under the influence of TME of immunosuppression, metabolic pathways such as activation of glycolysis, increase of lipid metabolism, and enhancement of mitochondrial biosynthesis reshape the local TME, and immune cells trigger metabolic adaptation through metabolic reprogramming to meet their own needs and play the role of anti-tumor or immunosuppression (27–29). The response of immune cells to tumor cells mainly depends on their specific metabolic pathway, which is related to the type and function of immune cells. In the resting state, naive T cells receive most of their energy through fatty acid oxidation, oxidative phosphorylation and glutamine metabolism. Upon activation, the effector T cells need to grow and proliferate rapidly and acquire the corresponding effector functions. To meet these demands, they upregulate the intensity of glycolysis, inhibit fatty acid oxidation, without obvious changes in oxidative phosphorylation levels to increase nutrient intake in an anabolic mode (**Figure 2**). Mechanistically, these metabolic changes may involve the activation of transcription factors such as HIF1α and c-Myc by TCR, CD28 and PI3K-AKT-mTOR pathways, which in turn upregulate the expression of glucose transport proteins, related metabolic enzymes (e.g. hexokinase 2 in glycolysis) and amino acid transport proteins (e.g. solute carrier family 5 member 5) (42).

Recently, a growing number of studies have confirmed the defection of immune cells that originally held anti-tumor activity in the immune microenvironment (43–45). However, considering that rapidly proliferating tumor cells compete with immune cells for relatively scarce nutrients, immune cells are in a state of nutritional deficiency due to lack of Warburg effect ability. Therefore, is the transformation of immune cells from defenders with tumor-killing capacity to enablers with immunosuppressive effects an active process stimulated by immune cells to obtain nutrients and energy? Is the depletion or loss of T cell tumor killing function a side effect of T cells competing for nutrients and thus evolving to protect themselves? In other words, whether T cells rely on the nutrition provided by cancer cells, thus giving up the ability to kill tumor cells? If these questions are valid, then it is reasonable to speculate that in the process of metabolic reprogramming of immune cells in response to nutritional stress, immune cells modify their original molecular phenotype through genetic reprogramming or epigenetic regulation, resulting in alterations in the mode of action and molecular mechanism of immune cells in response to tumor cells, which may explain why T cells in the TAM are always in a state of weakened tumor-killing capacity.

### Effects of fatty acid metabolism on T cell function

Numerous studies have shown that six key metabolic pathways [glycolysis, tricarboxylic acid cycle (TCA), pentose phosphate pathway, fatty acid oxidation, fatty acid synthesis and amino acid pathway] are involved in the organism’s immune response and its regulation through sophisticated mechanisms (46, 47). The different metabolic pathways are closely linked by a number of common metabolic intermediates. For example, fatty acid synthesis provides cell membranes and other critical lipid cell structures, while the raw material for its synthesis is derived from intermediates of the glycolytic pathway and TCA cycle metabolism. Intracellular signaling pathways tightly connect the activity of these metabolic pathways to the proliferation, activation, and differentiation of immune cells, thereby influencing the local and overall response of the immune system.

Fatty acid oxidation (FAO) takes place in mitochondria or peroxisomes and produces many metabolic intermediates with important physiological functions, including acetyl coenzyme A, nicotinamide adenine dinucleotide (NADHN) and reduced flavin adenine dinucleotide (FADH2), and large amounts of ATP (48). The fatty acid synthesis pathway is a process of intracellular lipid synthesis, which is required for cell growth and proliferation. The mTOR pathway regulates the expression and activity of many key enzymes in the *de novo* fatty acid synthesis pathway, including sterol regulatory element-binding transcription factor (SREBP), fatty acid synthase (FASN) and acetyl-Co A carboxylase (ACC) (49). Cell membranes and other key lipid cell structures essential for T cell proliferation depend on fatty acid synthesis, thus, induction of *de novo* fatty acid synthesis is essential for development and differentiation of effector T cells. For detailed information, please refer to (50). Notably, different types of immune cells exhibit different fatty acid metabolism patterns, such as M2 macrophages, Treg cells and memory T cells, which show a dependence on FAO, probably due to these cells live in a relatively nutrient-deficient microenvironment, making it more important to generate more ATP through FAO and to maintain normal mitochondrial function.

### Advantages of immunotherapy with γδ T cells

Activated γδT cells can exert anti-cancer effects through potent cytotoxicity, while preserving normal tissues (7). The non-MHC molecule-dependent recognition and killing of tumor cells and the high expression of NKG2D (an activating immune receptor expressed by NK and effector T cells), which are unique to γδ T cells, give them a broad spectrum of tumor cell killing. Secondly, γδ T cells play an important role in immunotherapy because of their ability to achieve massive expansion *in vivo* and *in vitro* (51). It has been confirmed that adoptive transfer of ex vivo expanded Vγ9Vδ2 T cells in combination with zoledronic acid inhibits cancer growth and limits osteolysis in a murine model of osteolytic breast cancer (52). The advantages of broad spectrum, high efficiency and no obvious side effects of γδ T cells can not only kill tumor cells efficiently to prevent their recurrence, but also reduce the occurrence of side effects when used in combination with radiotherapy, which provides a better guarantee for the survival quality of patients. Immune memory is a defining feature of the acquired immune system, but activation of the innate immune system can also result in enhanced responsiveness to subsequent triggers. This process has been termed ‘trained immunity’ (53). Recently, a randomized, placebo-controlled trial revealed a trained immunity program characterized by modulation of γδ T cell function, with higher production of TNF and IFN-γ, as well as upregulation of cellular metabolic pathways, induced by the measles, mumps, and rubella (MMR) vaccination, providing new idea for the future development of tumor vaccines (54). γδ T-cells exhibit bona fide tissue-residency in human liver and HCC, with tissue-resident memory γδ T-cells showing no egress from hepatic vasculature, persistence for >10 years and superior anti-tumor cytokine production (55). Thus, more universally effective HCC immunotherapy may be achieved by combining drugs (such as aminobisphosphonates) to induce tissue-resident memory γδ T-cells capable of replenishing the depleted pool, with additional intratumoural delivery to sensitize HCC to tissue-resident memory γδ T-cells-based targeting. Moreover, The use of peripheral γδ T lymphocyte cells in combination with the albumin-bilirubin scores are helpful for predicting the advanced HCC patients’ responses to immune checkpoint inhibitors (56). Furthermore, CD69+ Vδ1γδ T cells are described as functional Vδ1γδ T cell subsets in patients with HCC, and circulating CD69+ Vδ1γδ T cell is a promising candidate in immunotherapy of HCC (57). These results reveal the significant potential of γδ T cells in the immunotherapy of HCC. Nevertheless, there are still individual differences in HCC patients (58). Immunotherapy with γδ T cells needs to be further confirmed by animal and clinical experiments.

## A summary of our hypothesis

**Figure 3:**
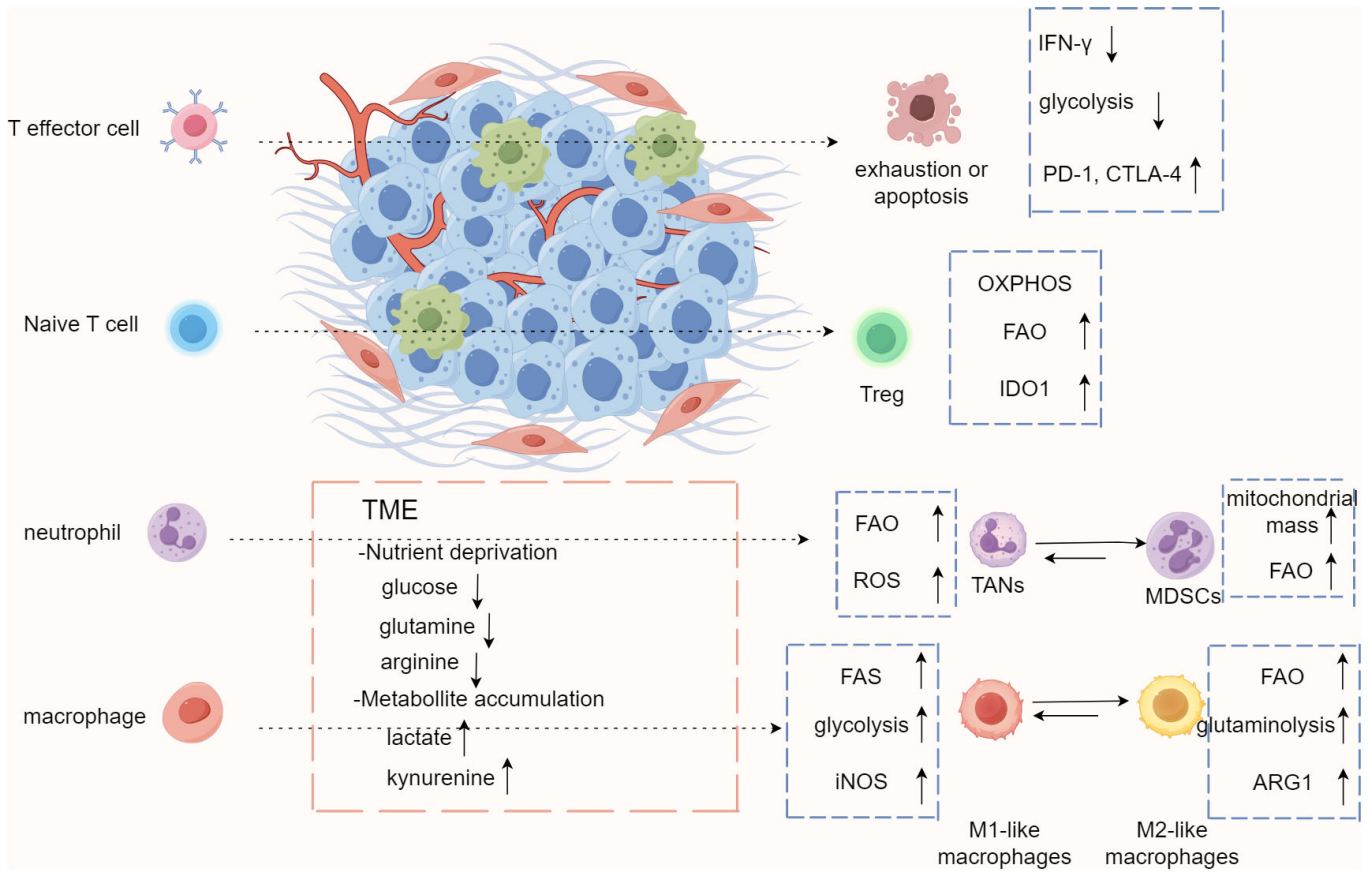
Immune cells trigger metabolic adaptation through metabolic reprogramming in tumor microenvironment. The conditions of tumor microenvironment (TME), including nutrient competition, low pH, limited oxygen, and accumulation of metabolites, result in immunosuppressive or tolerogenic phenotypes of immune cells and encourage metabolism that relies more on oxidative phosphorylation and fatty acid oxidation to fulfill energy needs. These conditions also promote differentiation and accumulation of Treg, M2-like macrophages, and MDSCs, which drives an immunosuppressive microenvironment generally. MDSCs, myeloid-derived dendritic cells; TAN, tumor-associated neutrophils.

T cells exhibit different molecular phenotypes at different tumor stages: initially tumor surveillance is coordinated by IFN-γ producing and cytotoxic γδ T cell subsets, but once the tumor grows, it is infiltrated by IL-17^+^ γδ T cell subsets that facilitate its expansion (11, 59). Whether this phenomenon suggest that γδ T cells in the TME may undergo epigenetic regulations or gene rearrangements that allow them to generate alternative molecular phenotypes needs to be further explored. Recent studies have revealed that metabolic pathways such as activation of glycolysis, increase of lipid metabolism, enhancement of mitochondrial biosynthesis, and alterations of fatty acid synthesis reshape the local TME, and immune cells trigger metabolic adaptation through metabolic reprogramming to meet their own needs and play the role of anti-tumor or immunosuppression (**Figure 3**). Metabolic reprogramming involves genome editing or epigenetic modifications. Consequently, in order to adapt to nutritional stress, T cells may lose their ability to kill tumor cells by altering their molecular phenotype to adapt to the hypoxic microenvironment. Combining previous studies and our bioinformatics results, we hypothesize that γδT cells compete for resources with HCC cells by means of fatty acid metabolic regulation in the TME, which results in the weakening or loss of their ability to recognize and kill HCC cells through genetic and epigenetic alterations, thus allowing γδT cells to be hijacked by HCC cells as a subpopulation that promotes HCC progression (**Figure 2**).

## Discussion

T-cell subsets are numerous and play an irreplaceable role in the immune response. The metabolic pattern of each subpopulation is different; therefore, elucidating the interrelationship between T cell metabolism and function will not only deepen the basic study of immune metabolism, but also provide potential targets for drug development and new strategies for clinical diagnosis and treatment. The non-MHC-restricted recognition of antigens by γδT cells can resist the escape of tumor cells, and the broad-spectrum killing of tumor cells and powerful and comprehensive immune effects have attracted widespread attention in the immunotherapy of cancers.

In this research, we summarized that tumor cells have the ability to shape the microenvironment of immunosuppression, regulate the metabolism of immune cells, further affect the development, differentiation and function of immune cells, and greatly limit the anti-tumor immune activity, by means of nutritional competition, secretion of cytokines and release of metabolites. In response to this starvation dilemma, immune cells may also reduce energy consumption in order to survive, and give up the ability to kill tumor cells through epigenetics or reprogramming of gene expression. Therefore, it is particularly important to study the mechanism that immune cells obtain enough energy through specific metabolic pathways to maintain their anti-tumor activity in the TME. There are certain limitations to the present study. First, the current studies on γδ T cells in the HCC microenvironment are very limited, and we were unable to obtain additional datasets for validation to increase the reliability and generalizability of our hypothesis. Furthermore, we propose a leap of conjecture based on the results of enrichment analysis of γδ T cells extracted from the HCC microenvironment and paracancerous tissues, which requires further proof from subsequent experiments. Thus, there is a considerable lack of the detailed descriptions of the critical steps in metabolic reprogramming process that leads to functional changes in γδ T cells in HCC development. Based on the research of TME metabolism, we can focus on the metabolic needs of immune cells in the immunosuppressed TME, so as to transform the immune response from the tumor-promoting type to the tumor-inhibiting type; meanwhile, combined with anti-tumor and multi-target immunotherapy drugs can avoid adaptive drug resistance and significantly improve the prognosis and survival of tumor.

## Data Availability

The original contributions presented in the study are included in the article/supplementary material. Further inquiries can be directed to the corresponding author.
